# Influence of acute dietary nitrate supplementation timing on nitrate metabolism, central and peripheral blood pressure and exercise tolerance in young men

**DOI:** 10.1007/s00421-023-05369-z

**Published:** 2023-12-02

**Authors:** Samantha N. Rowland, Lewis J. James, Emma O’Donnell, Stephen J. Bailey

**Affiliations:** 1 School of Sport, Exercise and Health Sciences, Loughborough University, Loughborough, LE11 3TU UK; 2 Department of Cardiovascular Science, University of Leicester, Leicester, UK

**Keywords:** Nitric oxide, Beetroot juice, Circadian rhythms, Cardiovascular health, Exercise performance

## Abstract

**Purpose:**

Dietary nitrate (NO_3_^−^) supplementation can lower systolic blood pressure (SBP) and improve exercise performance. Salivary flow rate (SFR) and pH are key determinants of oral NO_3_^−^ reduction and purported to peak in the afternoon. We tested the hypotheses that NO_3_^−^-rich beetroot juice (BR) would increase plasma [nitrite] ([NO_2_^−^]), lower SBP and improve exercise performance to a greater extent in the afternoon (AFT) compared to the morning (MORN) and evening (EVE).

**Method:**

Twelve males completed six experimental visits in a repeated-measures, crossover design. NO_3_^−^-depleted beetroot juice (PL) or BR (~ 13 mmol NO_3_^−^) were ingested in the MORN, AFT and EVE. SFR and pH, salivary and plasma [NO_3_^−^] and [NO_2_^−^], brachial SBP and central SBP were measured pre and post supplementation. A severe-intensity exercise tolerance test was completed to determine cycling time to exhaustion (TTE).

**Results:**

There were no between-condition differences in mean SFR or salivary pH. The elevation in plasma [NO_2_^−^] after BR ingestion was not different between BR-MORN, BR-AFT and BR-EVE. Brachial SBP was unchanged following BR supplementation in all conditions. Central SBP was reduced in BR-MORN (− 3 ± 4 mmHg), BR-AFT (− 4 ± 3 mmHg), and BR-EVE (− 2 ± 3 mmHg), with no differences between timepoints. TTE was not different between BR and PL at any timepoint.

**Conclusion:**

Acute BR supplementation was ineffective at improving TTE and brachial SBP and similarly effective at increasing plasma [NO_2_^−^] and lowering central SBP across the day, which may have implications for informing NO_3_^−^ supplementation strategies.

## Introduction

Diurnal variation in acute cardiovascular events is well established, with epidemiological data revealing increased incidence of strokes (Elliot 1998; Sheppard et al. 2015), myocardial infarctions (Cohen et al. 1997; Fabbian et al. 2017), and sudden cardiac death (Cohen et al. 1997) in the morning. Typical morning behaviours, including arousal from sleep, sudden postural changes, increased activity and psychological stress, instigate increases in sympathetic tone, vasoconstriction and peripheral arterial resistance, which contribute to the ‘morning surge’ in blood pressure (BP) (Kario 2010). Both peripheral and central BP exhibit parallel circadian rhythms, with lower values manifesting during night-time sleep, followed by abrupt increases with morning wakening before attaining peak values in the late afternoon (Douma and Gumz 2018). Diurnal fluctuations in exercise performance across a range of sport and exercise settings is also well established, with performance purported to attain peak and nadir levels in the afternoon and morning, respectively (Chtourou et al. 2011, 2012; Hammouda et al. 2012; Lericollais et al. 2009; Martin et al. 1999; Souissi et al. 2004; Hill 2014). Although BP and exercise performance exhibit a diurnal variation, the efficacy of dietary interventions to modulate these diurnal responses is unclear.

Dietary nitrate (NO_3_^−^) supplementation has been reported to improve various aspects of cardiovascular function, including lowering resting brachial BP and arterial stiffness variables, and to improve exercise performance (Jackson et al. 2018; Bahrami et al. 2021; Li et al. 2020; Senefeld et al. 2020). These effects have been linked to increased circulating plasma [nitrite] ([NO_2_^−^]), a substrate for nitric oxide (NO) production via the so-called NO_3_^−^–NO_2_^−^–NO pathway (Kapil et al. 2020). After ingestion, approximately 25% of NO_3_^−^ enters the enterosalivary circulation, being absorbed by, and concentrated in, the salivary glands on the first-pass (Govoni et al. 2008). Subsequently, NO_3_^−^-rich saliva is secreted into the oral cavity wherein NO_3_^−^ undergoes second-pass metabolism by anaerobic bacteria on the tongue which reduce salivary NO_3_^−^ to NO_2_^−^ (Doel et al. 2005; Duncan et al. 1995). Once swallowed, a portion of this NO_2_^−^ is reduced to NO and other reactive nitrogen intermediates in the stomach (Benjamin et al. 1994), with some NO_2_^−^ and reactive nitrogen intermediates entering systemic circulation for later NO generation (Kapil et al. 2020). Whilst NO_3_^−^ supplementation has the potential to improve BP, vascular function, and exercise performance, it is currently unclear whether such effects are consistent across the day.

The efficacy of the NO_3_^−^–NO_2_^−^–NO pathway to elicit physiological effects is dependent on NO_3_^−^ transport into the oral cavity and the host oral microbiome for NO_3_^−^ reduction (Govoni et al. 2008; Bailey et al. 2016; Hezel and Weitzberg 2015; Jansson et al. 2008; Lundberg 2012). Indeed, when salivary NO_3_^−^ uptake and secretion or oral NO_3_^−^ reduction are impaired, the increase in plasma [NO_2_^−^] and lowering in BP after NO_3_^−^ supplementation are attenuated (Govoni et al. 2008; Bailey et al. 2016; McDonagh et al. 2015). On the other hand, secretion of NO_3_^−^ into the oral cavity and exposure to the oral NO_3_^−^-reducing anaerobes will be enhanced by increasing salivary flow rate (SFR). However, although previous research has shown that music stimuli can elevate saliva secretion and salivary NO_2_^−^ generation (Jin et al. 2018), it is presently unclear whether diurnal variation in SFR impacts salivary and plasma [NO_3_^−^] and [NO_2_^−^].

In addition to SFR, salivary pH can impact oral NO_3_^−^ reduction. Specifically, increasing salivary pH after NO_3_^−^ supplementation has been reported to increase salivary and plasma [NO_2_^−^] (Cocksedge et al. 2023). Since both unstimulated SFR (Dawes 1975, 1972) and salivary pH (Choi et al. 2017; Ferguson and Fort 1974) exhibit circadian rhythms, with an acrophase in the afternoon, oral NO_3_^−^ reduction and the resultant increases in salivary and plasma [NO_2_^−^] after NO_3_^−^ supplementation may be greatest in the afternoon. Consistent with this postulate, oral NO_3_^−^ reduction was reported to be enhanced in the afternoon compared with the morning during a mouth rinse with a KNO_3_^−^ solution (Rowland et al. 2021). However, it is unclear whether enhanced oral NO_3_^−^ reduction in the afternoon during a brief mouth rinse is reproducible after NO_3_^−^ ingestion and whether this effect translates into greater plasma [NO_2_^−^], and more pronounced reductions in BP and improvements in exercise tolerance.

The purpose of this study was to investigate the effect of dietary NO_3_^−^ supplementation on NO_3_^−^ metabolism, peripheral and central BP, pulse wave variables, and exercise performance, and the extent to which any improvements in these variables after NO_3_^−^ supplementation exhibited a diurnal variation. It was hypothesised that NO_3_^−^ supplementation would increase salivary and plasma [NO_2_^−^], and improve brachial and central BP, pulse wave variables and exercise performance to a greater extent when ingested in the afternoon compared to the morning and evening.

## Methods

### Participants

Twelve young healthy males [mean ± SD: age: 23 ± 4 years, stature: 1.80 ± 0.09 m, body mass: 75.8 ± 10.9 kg, $\dot{V}{\text{O}}_{{{\text{2peak}}}}$: 50.4 ± 8.5 ml^.^kg^.^min^−1^, gas exchange threshold (GET): 118 ± 32 W, peak aerobic power (PAP): 323 ± 68 W] volunteered to participate in this study. None of the participants were tobacco smokers (Bailey et al. 2016) or vapers or taking any medication known to interfere with stomach acid production (e.g., proton pump inhibitors). No participants had any pre-existing medical conditions such as hypertension or diabetes. All participants were classified as recreationally active (McKay et al. 2022). Experimental testing was approved by Loughborough University Research Ethics Approvals Human Participants Sub Committee (ethics code: R18-P145) and confirmed with the principles of the Declaration of Helsinki, apart from registration in a database. Participants gave their written informed consent to participate.

### Pre-visit standardisation

Participants recorded their dietary intake 24 h prior to their first session and were asked to replicate this before subsequent visits. Each participant was given a list of NO_3_^−^- and thiocyanate-rich foods (Dewhurst-Trigg et al. 2018) to abstain from eating 24 h before sessions and asked to avoid caffeine and alcohol ingestion in the 12 h and 24 h before each visit, respectively. All visits were conducted in a postprandial state. Since SFR is reduced in a state of hypohydration (Ship and Fischer 1997), participants were provided with 40 mL·kg^−1^ body mass^−1^ of fluid to consume in the 24 h before each visit (Minshull and James 2013) and instructed to consume 500 mL of water 1 h before testing to ensure euhydration on arrival. During testing sessions, participants were given 300 mL of water in 2 equal boluses to ensure euhydration was maintained. Since antibacterial mouthwash disrupts oral NO_3_^−^ reduction (Govoni et al. 2008), participants were required to abstain from using mouthwash 48 h prior to each testing session. Participants were instructed to maintain their habitual exercise patterns for the duration of the study but were required to avoid strenuous exercise in the 24 h prior to each visit.

### Experimental design

Participants reported to the laboratory on eight occasions. During the first visit, participants were familiarised with all the experimental procedures and completed a ramp incremental test for determination of GET, PAP and $\dot{V}{\text{O}}_{{{\text{2peak}}}}$. GET is a non-invasive estimate of the lactate threshold and demarcates the boundary between the moderate and heavy intensity exercise domains. PAP was the maximum power output attained during the incremental ramp test. $\dot{V}{\text{O}}_{{{\text{2peak}}}}$ is defined as the highest volume of oxygen uptake during the incremental ramp test. During visit two, participants were familiarised with the time to exhaustion (TTE) exercise protocol. In the six main experimental visits, baseline measures of SFR and pH, BP and vascular function (pulse wave analysis) were obtained, in sequence, and salivary and plasma samples were collected for later assessment of [NO_3_^−^] and [NO_2_^−^]. Urine and serum osmolality were also evaluated at baseline to assess hydration status. Subsequently, participants ingested 2 × 70 mL of concentrated NO_3_^−^-rich (BR; 13 mmol NO_3_^−^) or NO_3_^−^-depleted (PL; ~ 0.04 mmol NO_3_^−^) beetroot juice (Beet It, James White Drinks Ltd., Ipswich, UK) with 30 g of cornflakes and 125 mL of semi-skimmed milk. Saliva measurements were repeated 1 h post beetroot ingestion. All baseline measurements were then repeated 2.5 h following beetroot ingestion to coincide with the peak plasma [NO_2_^−^] (Wylie et al. 2013). Finally, participants completed the TTE exercise test. The six experimental conditions, PL and BR in the morning (started at 08:00; PL-MORN and BR-MORN), afternoon (started at 12:00; PL-AFT, BR-AFT) and evening (started at 15:00; PL-EVE and BR-EVE) were administered in a randomised, repeated-measures, crossover experimental design. PL and BR supplement administration was randomised (counterbalancing not possible due to the number of sequence permutations) and double-blinded (supplement bags labelled 1 and 2 by an independent investigator). Supplement ingestion occurred at 09:00, 13:00 and 16:00 in the MORN, AFT and EVE, respectively.

### Measurements

#### Hydration status

Urine samples obtained at baseline was analyzed immediately to evaluate urine osmolality (Osmocheck, Vitech Scientific, UK), with a reading of < 700 mOsmol/kg required for visit continuation (Sawka et al. 2007). Additionally, 5 mL of venous blood was collected into a serum tube and left to clot at room temperature, with serum separated by centrifugation (3500 × g at 4 °C for 15 min) and frozen at − 80 °C for later analysis of osmolality via freezing-point depression (Gonotec 225 Osmomat 030 Cryoscopic Osmometer; Gonotec, Germany). Serum osmolality values ranging between 285 and 295 mOsmol^.^kg H_2_O^−1^ were taken to imply euhydration (Knepper et al. 2015).

#### Saliva collection

Prior to sample collection, participants rinsed their oral cavity with room-temperature tap water to remove any food debris. Following 2 min rest, unstimulated saliva samples were collected via passive drool and spit into pre-weighed sterile containers every 20 s for 2 min. This process was then repeated after 2 min. Samples were subsequently weighed for determination of SFR before salivary pH was measured in duplicate using a microFET electrode (Sentron, Leek, The Netherlands), with the measured pH value accepted once readings on the pH meter were stable for 5 s. A 3-point calibration of the pH probe was undertaken prior to analysis using buffers with known pH (4.01, 7.00, 10.01). 1 mL aliquots were then frozen at − 80 °C for later analysis of salivary [NO_3_^−^] and [NO_2_^−^]. Given that salivary [NO_3_^−^] and [NO_2_^−^] are influenced by SFR (Granli et al. 1989), salivary [NO_3_^−^] and [NO_2_^−^] data were also normalised to SFR to report salivary [NO_3_^−^] and [NO_2_^−^] flux per min.

#### Blood pressure

Participants were required to rest supine for 10 min. Thereafter, BP of the brachial artery was measured using an automated sphygmomanometer (Omron Healthcare, Kyoto, Japan). In total, five measurements were taken at 2 min intervals, with the mean of all five readings used for analysis. MAP was calculated as ([(2 × DBP) + SBP]/3).

#### Aortic blood pressure and pulse wave variables

Following 20 min supine rest, pulse wave analysis was assessed at the radial artery using applanation tonometry methods (SphygmoCor; Atcor Medical, Sydney, Australia) to determine central BP and indices of arterial stiffness. Pulse wave analysis calibrated to brachial BP uses a validated generalised transfer function to derive corresponding central aortic pressures (Chen et al. 1997). All tonometry data were recorded by a single investigator. A minimum of two recordings were taken at each time interval and the two measurements with the highest quality index (> 80%) were accepted for analysis. Pulse wave analysis indices of interest included: aortic systolic and diastolic BP, augmentation pressure (AP: the amplitude of the reflected wave), augmentation index (AI: the reflected wave amplitude divided by pulse pressure expressed as a percentage) and, due to the known influence of HR on AI, AI adjusted for heart rate of 75 bpm (AI@HR75).

#### Blood collection

Following 30 min supine rest, a tourniquet was applied around the upper arm prior to sample collection. Blood samples were subsequently drawn from an antecubital vein via venepuncture into 6 mL lithium heparin vacutainers. Samples were centrifuged at 3000 × g and 4 °C for 10 min, within 2 min of collection. Plasma was subsequently extracted and immediately frozen at − 80 °C for later analysis of [NO_3_^−^] and [NO_2_^−^].

#### Exercise procedures

All exercise tests were performed on an electronically-braked cycle ergometer (Lode Excalibur Sport, Groningen, The Netherlands). During the first laboratory visit, participants completed a ramp incremental test involving 4 min of baseline cycling at 20 W followed by a linear 30 W/min increase in work rate until task failure. Task failure was recorded once the pedal rate fell ≥ 10 rpm below self-selected cadence (70–100 rpm) for ≥ 5 s. The saddle and handlebar height and configuration were recorded and reproduced in subsequent tests. Breath-by-breath pulmonary gas exchange data were collected continuously during the incremental test and averaged over consecutive 10 s periods (Vyntus CPX metabolic cart, Vyaire Medical, Chicago, USA). Participants wore a face mask and breathed through a low dead space, low resistance, digital volume transducer assembly. The inspired and expired gas volume and gas concentration signals were continuously sampled via a capillary line connected to the mouthpiece. The gas analyser was calibrated prior to testing with gases of known concentration. The turbine volume transducer was calibrated automatically and manually using a 3 L syringe (Hans Rudolph, Kansas City, Missouri). $\dot{V}{\text{O}}_{{{\text{2peak}}}}$ was taken as the highest 30 s mean value attained prior to the participant’s volitional exhaustion. GET, was determined from a cluster of measurements including (1) the first disproportionate increase in CO_2_ production ($\dot{V}{\text{CO}}_{{\text{2}}}$) from visual inspection of individual plots of $\dot{V}{\text{CO}}_{{\text{2}}}$ vs. $\dot{V}{\text{O}}_{{\text{2}}}$, (2) an increase in expired ventilation ($\dot{V}{\text{E}}$)/$\dot{V}{\text{O}}_{{\text{2}}}$ with no increase in $\dot{V}{\text{E}}$/$\dot{V}{\text{CO}}_{{\text{2}}}$, and (3) an increase in end-tidal O_2_ tension with no fall in end-tidal CO_2_ tension. The TTE protocols involved 4 min cycling at 20 W followed by a step increase in work rate equivalent to 75%Δ (GET + 75% of the difference between the work rate at GET and PAP), with account taken for the mean response time for $\dot{V}{\text{O}}_{{\text{2}}}$ during the ramp protocol (i.e., two-thirds of the ramp rate (20 W) deducted from the work rate at GET and PAP to account for the muscle-to-lung gas transit time). The test was terminated once pedal cadence fell ≥ 10 rpm below the self-selected cadence for ≥ 5 s. This exercise protocol was replicated during visits 3–8 and TTE was recorded.

#### [NO_3_^−^] and [NO_2_^−^] determination

All glassware, utensils and surfaces were rinsed thoroughly with deionised water to remove residual NO_3_^−^ and NO_2_^−^ prior to analysis. Plasma samples were deproteinised prior to [NO_3_^−^] determination. Firstly, 500 μL of 0.18 N NaOH was added to 100 µL of sample followed by 5 min incubation at room temperature. Subsequently, samples were treated with 300 μL aqueous ZnSO4 (5% w/v) and vortexed for 30 s before undergoing an additional 10 min incubation period at room temperature. Samples were then centrifuged at 21,000 × g for 10 min and the supernatant was removed for subsequent analysis. The [NO_3_^−^] of the deproteinised plasma sample was determined by its reduction to NO in the presence of 0.8% (w/v) vanadium chloride (VCl_3_) in 1 M HCl via 50 μL injections into the septum of the air-tight purge vessel. The spectral emission of electronically excited nitrogen dioxide, derived from the reaction of NO with ozone, was detected by a thermoelectrically cooled, red-sensitive photomultiplier tube housed in a gas-phase chemiluminescence NO analyser (Sievers NOA 280i, Analytix Ltd, Durham, UK). All samples were analyzed in duplicate. The [NO_3_^−^] was determined by plotting signal (mV) area against a calibration plot of sodium nitrate standards. Prior to plasma [NO_2_^−^] determination, samples were deproteinised using ice-cold ethanol. Specifically, 500 μL of ethanol was added to 500 μL of sample followed by 15 min incubation. Samples were then centrifuged at 21,000 × g for 10 min and the supernatant was removed for subsequent analysis. Plasma [NO_2_^−^] was determined by its reduction to NO in the presence of glacial acetic acid and aqueous sodium iodide (4% w/v) and calibrated using sodium nitrite standards. To determine plasma [NO_2_^−^], 200 μL of deproteinised plasma was injected into the purge vessel. Origin Lab was used to smooth the NO analyser signal and objectively identify the peaks to derive the NO_2_^−^ concentration data. After thawing at room temperature, saliva samples were centrifuged for 10 min at 21,000 × g and the supernatant was then removed and diluted at least 100-fold with deionised water for subsequent analysis. [NO_3_^−^] and [NO_2_^−^] were determined using the same reagents described above for the respective plasma analyses.

### Statistical analysis

Statistical analysis was performed using IBM SPSS Statistics version 27. Shapiro Wilk’s test was used to check data normality. Baseline data and data containing one factor (condition [PL-MORN, BR-MORN, PL-AFT, BR-AFT, PL-EVE, BR-EVE] including hydration biomarkers, SFR, salivary pH, and TTE) were analyzed using one-way repeated-measures ANOVAs. Plasma [NO_3_^−^] and [NO_2_^−^], BP and vascular function were initially analysed using two-way repeated-measures ANOVAs (condition [PL-MORN, BR-MORN, PL-AFT, BR-AFT, PL-EVE, BR-EVE] × time [0 h and 2.5 h]). Salivary [NO_3_^−^] and [NO_2_^−^] were initially analyzed using two-way repeated-measures ANOVAs (condition [PL-MORN, BR-MORN, PL-AFT, BR-AFT, PL-EVE, BR-EVE] × time [0 h, 1 h, 2.5 h]). Significant ANOVA interaction effects were followed up with post hoc Dunnett’s tests for comparisons to baseline control for the salivary data and Holm-Bonferroni corrected paired-samples *t* tests were used for all other variables. To calculate effect sizes, partial eta squared (*n*_*p*_^*2*^) was used for the omnibus tests and Cohen’s *d*_z_ (t/√n) for paired-samples *t* tests. All data are displayed as mean ± SD unless otherwise stated. Statistical significance was accepted at *P* ≤ 0.05.

## Results

### Hydration biomarkers

For all participants, urine osmolality on arrival was < 700 mOsmol^.^kg H_2_O^−1^ in all six conditions. Serum osmolality was between 285 and 295 mOsmol kg H_2_O^−1^ across all visits and not different between conditions (*P* > 0.050, *n*_*p*_^*2*^ = 0.03).

### Salivary flow rate and pH

**Fig. 1:**
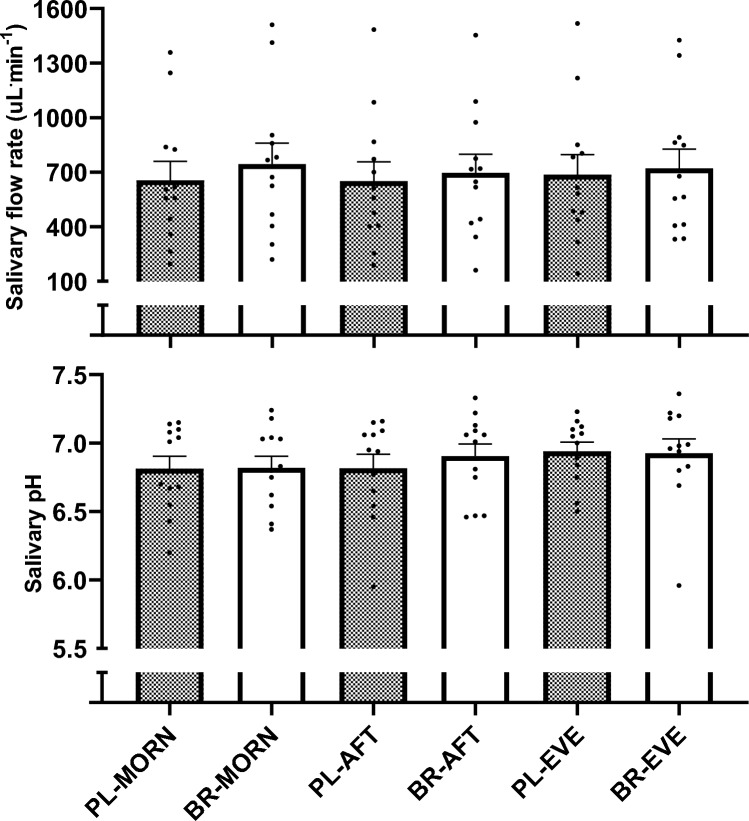
Mean salivary flow rate (SFR; upper panel) and salivary pH (lower panel) from 1 to 2.5 h following ingestion of nitrate-depleted and nitrate-rich beetroot juice in the morning (PL-MORN and BR-MORN), afternoon (PL-AFT and BR-AFT) and evening (PL-EVE and BR-EVE). The bars represent the group mean ± SEM responses with the filled circles representing individual participants. No differences observed between conditions (*P* > 0.050)

There were no inter-condition differences in SFR or salivary pH at baseline (both *P* > 0.050). Mean SFR and pH between 1 and 2.5 h did not differ between conditions (*n*_*p*_^*2*^ = 0.14, *n*_*p*_^*2*^ = 0.16, both *P* > 0.050, Fig. 1, respectively).

### Salivary [NO_3_^−^] and [NO_2_^−^]

**Fig. 2:**
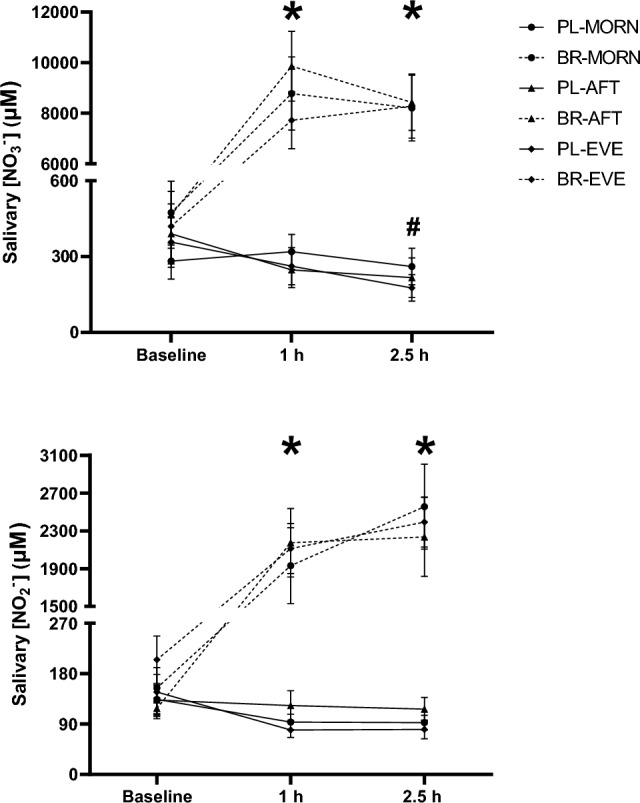
Salivary nitrate concentration ([NO_3_^−^], upper panel) and salivary nitrite concentration ([NO_2_^−^], lower panel) at baseline, 1 h and 2.5 h following ingestion of nitrate-depleted and nitrate-rich beetroot juice in the morning (PL-MORN and BR-MORN), afternoon (PL-AFT and BR-AFT) and evening (PL-EVE and BR-EVE). Data presented as the group mean ± SEM responses with the filled circles representing individual participants. *Denotes higher than PL-MORN, PL-AFT and PL-EVE in BR-MORN, BR-AFT and BR-EVE (*P* < 0.050). #denotes salivary [NO_3_^−^] lower than baseline at 2.5 h in PL-AFT and PL-EVE (*P* < 0.050)

There were no inter-condition differences in salivary [NO_3_^−^] at baseline (*P* > 0.050). There was a main effect for condition (*P* < 0.001, *n*_*p*_^*2*^ = 0.79) and time (*P* < 0.001, *n*_*p*_^*2*^ = 0.84), and a condition × time interaction effect (*P* < 0.001, *n*_*p*_^*2*^ = 0.71) for salivary [NO_3_^−^]. Compared to baseline, salivary [NO_3_^−^] was unchanged at 1 h and 2.5 h in PL-MORN (*P* > 0.050) but reduced at 2.5 h vs baseline in PL-AFT (*P* = 0.034) and PL-EVE (*P* = 0.018). Salivary [NO_3_^−^] was elevated above baseline at all time points in BR conditions (all *P* < 0.001), with no differences between BR-MORN, BR-AFT and BR-EVE at 1 h (*d*_z_ ≤ 0.60) or 2.5 h (*d*_z_ ≤ 0.05, both *P* > 0.050, Fig. 2). Normalising salivary [NO_3_^−^] relative to SFR did not alter any of the observed effects compared to absolute salivary [NO_3_^−^].

There were no inter-condition differences in salivary [NO_2_^−^] at baseline (*P* > 0.050). There was a main effect for condition (*P* < 0.001, *n*_*p*_^*2*^ = 0.74) and time (*P* < 0.001, *n*_*p*_^*2*^ = 0.79), and a condition × time interaction effect (*P* < 0.001, *n*_*p*_^*2*^ = 0.66) for salivary [NO_2_^−^]. Salivary [NO_2_^−^] was unchanged between 0 and 2.5 h in PL-MORN, PL-AFT and PL-EVE (all *P* > 0.050). Salivary [NO_2_^−^] was increased above baseline at all time points in BR-MORN, BR-AFT and BR-EVE (all *P* < 0.001), with no inter-condition differences at 1 h (*d*_z_ ≤ 0.16) or 2.5 h (*d*_z_ ≤ 0.42, both *P* > 0.050, Fig. 2). Normalising salivary [NO_2_^−^] to SFR did not change any of the observed effects compared to absolute salivary [NO_2_^−^].

### Plasma [NO_3_^−^] and [NO_2_^−^]

**Fig. 3:**
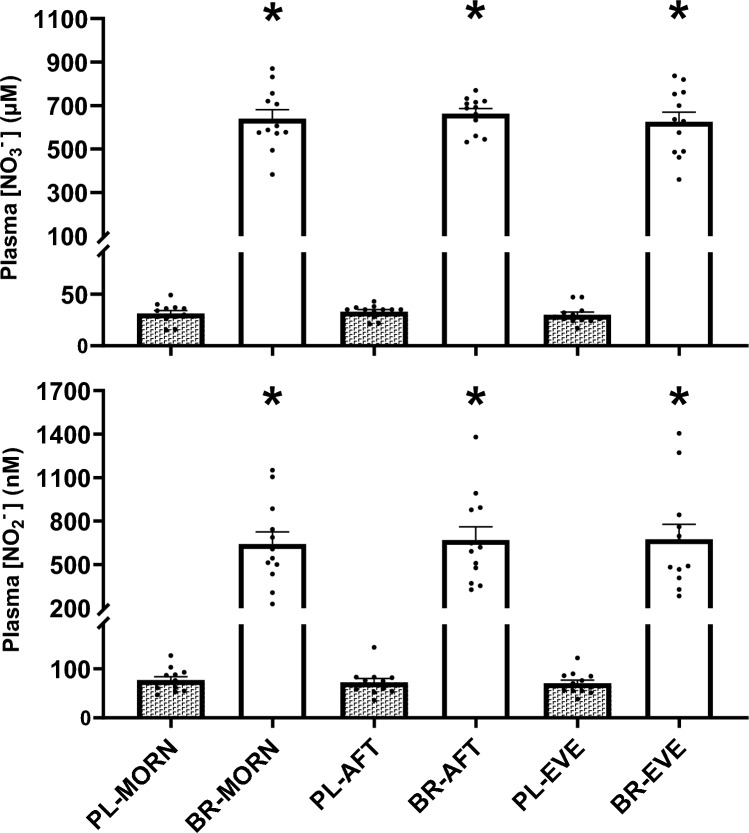
Plasma nitrate concentration ([NO_3_^−^], upper panel) and plasma nitrite concentration ([NO_2_^−^], lower panel) 2.5 h following ingestion of nitrate-depleted and nitrate-rich beetroot juice in the morning (PL-MORN and BR-MORN), afternoon (PL-AFT and BR-AFT) and evening (PL-EVE and BR-EVE). The bars represent the group mean ± SD responses with the filled circles representing individual participants. *Denotes higher than PL-MORN, PL-AFT and PL-EVE in BR-MORN, BR-AFT and BR-EVE (*P* < 0.050)

Plasma [NO_3_^−^] was not different between conditions at baseline (*P* > 0.050). There was a main effect for condition (*P* < 0.001, *n*_*p*_^*2*^ = 0.95) and time (*P* < 0.001, *n*_*p*_^*2*^ = 0.96), and a condition × time interaction effect (*P* < 0.001, *n*_*p*_^*2*^ = 0.92). Plasma [NO_3_^−^] remained stable between baseline and 2.5 h in PL-MORN, PL-AFT and PL-EVE (all *P* > 0.050) but was elevated above baseline at 2.5 h in BR-MORN (640 ± 141 µM), BR-AFT (663 ± 79 µM) and BR-EVE (626 ± 154 µM) (all *P* < 0.001), with no differences between the BR conditions (*P* > 0.050, *d*_z_ ≤ 0.31, Fig. 3).

Plasma [NO_2_^−^] was not different between conditions at baseline (*P* > 0.050). There was a main effect for condition (*P* < 0.001, *n*_*p*_^*2*^ = 0.73) and time (*P* < 0.001, *n*_*p*_^*2*^ = 0.80), and a condition × time interaction effect (*P* < 0.001, *n*_*p*_^*2*^ = 0.76). Plasma [NO_2_^−^] was unchanged between 0 and 2.5 h in PL-MORN (*P* > 0.050) but decreased in PL-AFT (*P* = 0.027) and PL-EVE (*P* = 0.050). Plasma [NO_2_^−^] was increased above baseline 2.5 h post supplement ingestion in BR-MORN (642 ± 289 nM), BR-AFT (670 ± 314 nM) and BR-EVE (675 ± 355 nM) (all *P* < 0.001), with no differences between conditions (*P* > 0.050, *d*_z_ ≤ 0.11, Fig. 3).

### Brachial artery blood pressure

#### Systolic blood pressure

There were no inter-condition differences in brachial SBP at baseline (*P* > 0.050). There was a main effect for time (*P* = 0.007, *n*_*p*_^*2*^ = 0.50), but no main effect for condition (*P* > 0.050, *n*_*p*_^*2*^ = 0.05) or condition × time interaction (*P* > 0.050, *n*_*p*_^*2*^ = 0.09, Table 1).

**Table 1:** Brachial artery blood pressure at baseline and 2.5 h following ingestion of nitrate-depleted or nitrate-rich beetroot juice in the morning, afternoon, and evening

	PL-MORN	BR-MORN	PL-AFT	BR-AFT	PL-EVE	BR-EVE
SBP (mmHg)						
Baseline	119 ± 7	119 ± 7	118 ± 6	119 ± 7	119 ± 7	119 ± 8
2.5 h	118 ± 9	115 ± 7	118 ± 6	117 ± 7	118 ± 7	118 ± 8
DBP (mmHg)						
Baseline	68 ± 7	67 ± 8	67 ± 6	68 ± 7	67 ± 8	67 ± 8
2.5 h	69 ± 7	67 ± 7	68 ± 7	67 ± 6	70 ± 7	69 ± 7
MAP (mmHg)						
Baseline	85 ± 6	85 ± 7	84 ± 6	85 ± 6	84 ± 7	84 ± 7
2.5 h	85 ± 7	83 ± 6	83 ± 9	83 ± 5	85 ± 7	85 ± 6

Brachial artery systolic blood pressure (SBP), diastolic blood pressure (DBP) and mean arterial pressure (MAP) at baseline and 2.5 h following ingestion of nitrate-depleted or nitrate-rich beetroot juice in the morning (PL-MORN and BR-MORN), afternoon (PL-AFT and BR-AFT) and evening (PL-EVE and BR-EVE). Data are presented as group mean ± SD. No differences observed between conditions (*P* > 0.050)

#### Diastolic blood pressure

There were no inter-condition differences in brachial DBP at baseline (*P* > 0.050). There was a condition × time interaction (*P* = 0.005, *n*_*p*_^*2*^ = 0.25), but no main effect for condition (*P* > 0.050, *n*_*p*_^*2*^ = 0.03) or time (*P* > 0.050, *n*_*p*_^*2*^ = 0.11). Follow up post-hoc analysis revealed that brachial DBP was unchanged over time in PL-MORN (*d*_z_ = 0.46), PL-AFT (*d*_z_ = 0.60) and PL-EVE (*d*_z_ = 0.67), and in BR-MORN (*d*_z_ = 0.08), BR-AFT (*d*_z_ = 0.51), and BR-EVE (*d*_z_ = 0.34, all *P* > 0.050, Table 1).

#### Mean arterial pressure

There were no inter-condition differences in brachial MAP at baseline (*P* > 0.050) or any main effects for condition (*P* > 0.050, *n*_*p*_^*2*^ = 0.05) or time (*P* > 0.050, *n*_*p*_^*2*^ = 0.02), or a condition × time interaction (*P* > 0.050, *n*_*p*_^*2*^ = 0.11, Table 1).

#### Aortic blood pressure and arterial stiffness

##### Central systolic blood pressure

There were no inter-condition differences in central SBP at baseline (*P* > 0.050). There was a main effect for time (*P* = 0.011, *n*_*p*_^*2*^ = 0.49) and condition × time interaction (*P* = 0.007,* n*_*p*_^*2*^ = 0.27) but no main effect for condition (*P* > 0.050, *n*_*p*_^*2*^ = 0.04). Central SBP was unchanged over time in PL-MORN (*d*_z_ = 0.09), PL-AFT (*d*_z_ = 0.19) and PL-EVE (*d*_z_ = 0.28, all *P* > 0.050). Central SBP was lower at 2.5 h compared to baseline within BR-MORN (*P* = 0.030, *d*_z_ = 0.88), BR-AFT (*P* = 0.009, *d*_z_ = 1.19) and BR-EVE (*P* = 0.046, *d*_z_ = 0.69), with no differences between these conditions (*P* > 0.050, *d*_z_ ≤ 0.44, Table 2).

**Table 2:** Central blood pressure and pulse wave analysis variables at baseline and 2.5 h following ingestion of nitrate-depleted or nitrate-rich beetroot juice in the morning, afternoon, and evening

	PL-MORN	BR-MORN	PL-AFT	BR-AFT	PL-EVE	BR-EVE
SBP (mmHg)						
Baseline	100 ± 6	100 ± 7	99 ± 6	101 ± 5	100 ± 6	101 ± 7
2.5 h	100 ± 8	97 ± 6*	100 ± 6	98 ± 6*	99 ± 6	99 ± 7*
DBP (mmHg)						
Baseline	69 ± 6	70 ± 7	69 ± 5	71 ± 5	69 ± 6	69 ± 5
2.5 h	71 ± 6	69 ± 6	70 ± 6	68 ± 5	71 ± 7	70 ± 7
AP (mmHg)						
Baseline	– 3 ± 3	– 2 ± 4	– 2 ± 3	– 2 ± 3	– 3 ± 2	– 1 ± 4
2.5 h	– 1 ± 4	– 3 ± 3	– 1 ± 4	– 3 ± 3*	– 3 ± 2	– 4 ± 2
AI (%)						
Baseline	– 10 ± 8	– 7 ± 13	– 8 ± 9	– 7 ± 10	– 10 ± 7	– 5 ± 11
2.5 h	– 6 ± 12	– 13 ± 9	– 4 ± 13	– 11 ± 10	– 11 ± 7	– 12 ± 7
AI@HR75 (%)						
Baseline	– 17 ± 9	– 14 ± 12	– 15 ± 9	– 15 ± 10	– 17 ± 9	– 12 ± 12
2.5 h	– 16 ± 11	– 20 ± 8	– 14 ± 12	– 20 ± 9	– 19 ± 7	– 20 ± 8

Central systolic blood pressure (SBP), central diastolic blood pressure (DBP), augmentation pressure (AP), augmentation index (AI) and augmentation index normalised to heart rate (AI@HR75) at baseline and 2.5 h following ingestion of nitrate-depleted or nitrate-rich beetroot juice in the morning (PL-MORN and BR-MORN), afternoon (PL-AFT and BR-AFT) and evening (PL-EVE and BR-EVE). Data are presented for *n* = 11 as group mean ± SD

*Denotes lower than baseline (*P* < 0.050)

##### Central diastolic blood pressure

There were no inter-condition differences in central DBP at baseline (*P* > 0.050). There was no main effect for condition (*P* > 0.050, *n*_*p*_^*2*^ = 0.02) or time (*P* > 0.050, *n*_*p*_^*2*^ = 0.02), but there was a condition × time interaction effect (*P* = 0.011, *n*_*p*_^*2*^ = 0.25). Post hoc analysis revealed no significant differences between conditions or over time (*P* > 0.050, Table 2).

##### Augmentation pressure, augmentation index and augmentation index normalised to heart rate

There were no inter-condition differences in AP, AI or AI@HR75 at baseline (all *P* > 0.050). There was no main effect for condition (*P* > 0.050, *n*_*p*_^*2*^ = 0.06) or time (*P* > 0.050, *n*_*p*_^*2*^ = 0.05) but there was a significant condition × time interaction (*P* = 0.029, *n*_*p*_^*2*^ = 0.22) for AP. AP was lower at 2.5 h vs baseline in BR-AFT (*P* = 0.045, *n*_*p*_^*2*^ = 0.89), but no differences were observed in the other conditions (all *P* > 0.050, Table 2). There was no main effect for condition (*P* > 0.050, *n*_*p*_^*2*^ = 0.08) or time (*P* > 0.050, *n*_*p*_^*2*^ = 0.17) for AI; however, there was a condition × time interaction (*P* = 0.022, *n*_*p*_^*2*^ = 0.23). Post hoc analysis revealed no significant differences between conditions (*P* > 0.050, Table 2). There was a main effect for time (*P* = 0.024, *n*_*p*_^*2*^ = 0.42) for AI@HR75, but no main effect for condition (*P* > 0.050, *n*_*p*_^*2*^ = 0.04) or condition × time interaction (*P* > 0.050, *n*_*p*_^*2*^ = 0.19, Table 2).

##### Exercise performance

**Fig. 4:**
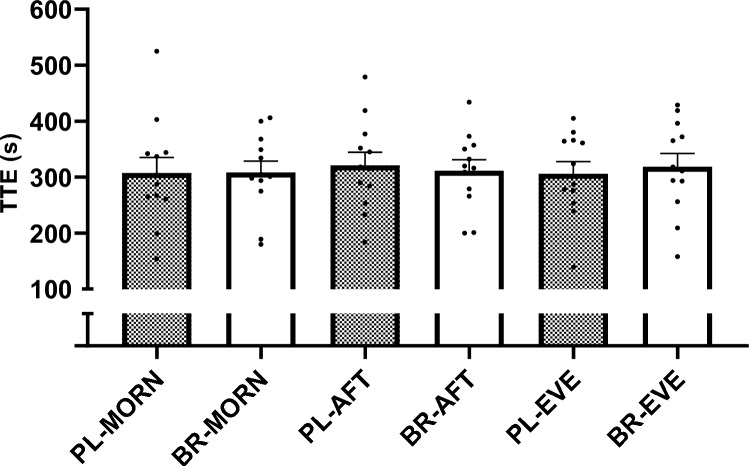
Time to exhaustion (TTE) during severe-intensity cycling exercise following ingestion of nitrate-depleted or nitrate-rich beetroot juice in the morning (PL-MORN and BR-MORN), afternoon (PL-AFT and BR-AFT) and evening (PL-EVE and BR-EVE) (upper panel). The bars represent the group mean ± SEM responses with the filled circles representing individual participants. No differences observed between conditions (*P* > 0.050)

Exercise TTE did not differ between PL-MORN (307 ± 96 s), BR-MORN (308 ± 71 s), PL-AFT (321 ± 81 s), BR-AFT (311 ± 68 s), PL-EVE (306 ± 76 s) and BR-EVE (318 ± 83 s), (*P* > 0.050, *n*_*p*_^*2*^ = 0.02; Fig. 4).

## Discussion

This study assessed whether the time-of-day an acute dose of dietary NO_3_^−^ was administered influenced its efficacy to lower BP and improve exercise performance in healthy adults. The principal novel findings from this study were: (1) SFR, salivary pH, BP and exercise performance did not exhibit a marked circadian rhythm; (2) salivary and plasma [NO_3_^−^] and [NO_2_^−^] were increased by a similar magnitude after BR ingestion in the morning, afternoon and evening; (3) BR consumption lowered central SBP by a similar magnitude across the day but did not reduce brachial SBP; and (4) severe-intensity cycling TTE was not improved with BR supplementation irrespective of the time of day ingested. These findings improve understanding of the effects of acute BR supplementation on BP, vascular function and exercise performance in healthy young men by evaluating the potential for time-specific effects of BR supplementation on these health indices.

### Salivary flow rate and pH

Previous studies have shown both unstimulated SFR (Dawes 1975, 1972) and salivary pH (Choi et al. 2017; Ferguson and Fort 1974) exhibit circadian variability, being lowest during sleep and the early morning and peaking mid-afternoon. Contrary to previous findings, SFR and salivary pH did not exhibit a circadian rhythm in the current study with these variables not being significantly different across the morning, afternoon, and evening assessment points. It is well documented that food and fluid consumption can alter SFR and salivary pH (Ship and Fischer 1997; Brunstrom et al. 2000; Watanabe and Dawes 1988). Since fluid consumption and food intake was standardised over the 24 h preceding each testing session and participants were objectively determined to be euhydrated in the current study, the lack of diurnal variation on SFR and salivary pH is unlikely to be a result of altered dietary intake. However, it is plausible that our hydration protocol may have overridden the underlying daily rhythm in SFR. Moreover, both SFR and salivary pH were highly variable between-participants, which likely impeded the detection of any subtle changes in these variables across the day. We concede that a larger sample size may have been necessary to detect subtle changes in SFR and salivary pH. It should also be acknowledged that the methods used to collect and measure SFR and pH may not have been sufficiently sensitive to detect small diurnal variability in these responses. Previous studies have used passive drool techniques or fitted oral collection devices to collect saliva and have collected samples for 11–12 successive days between ~ 07:00 and 22:00 to evaluate SFR (Dawes 1975, 1972). Similarly, a previous study reporting circadian-like patterns in salivary pH captured continuous changes over 48 h using custom-made intraoral appliances (Choi et al. 2017). In contrast, the current study only measured SFR and salivary pH on nine occasions between 08:00 and 18:30 and with different techniques, which may account for the lack of a significant within-day variability in SFR or salivary pH herein.

### Dietary nitrate metabolism

Salivary and plasma [NO_3_^−^] and [NO_2_^−^] were not different between pre-supplementation baseline measures during the morning, afternoon, and evening experimental testing sessions. Consistent with previous research (Bailey et al. 2016; Cocksedge et al. 2023; Burleigh et al. 2018; Woessner et al. 2016), both salivary and plasma [NO_3_^−^] and [NO_2_^−^] increased following the acute ingestion of BR in the present study. However, contrary to the experimental hypothesis, the increases in salivary and plasma [NO_3_^−^] and [NO_2_^−^] after BR supplementation were consistent across the morning, afternoon, and evening. These findings align with previous research which has shown stability in plasma and urinary [NO_3_^−^] (Ringqvist et al. 2000) and plasma [NO_x_] (Tangphao et al. 1999) over a 24 h period. The lack of diurnal variation in the evaluated NO_3_^−^ metabolism biomarkers in the current study may be partially attributed to the absence of a circadian rhythm in SFR and salivary pH. However, it is worth acknowledging that there may be diurnal variation in salivary and plasma [NO_3_^−^] and [NO_2_^−^] if assessed using more ecologically valid experimental designs (i.e., where individuals maintain their habitual dietary intake and activity levels) due to the NO_3_^−^ content of the diet with different meals.

### Blood pressure and vascular function

Despite evidence of circadian rhythms in BP reported in the literature (Douma and Gumz 2018; Williams et al. 2013; Jankowski et al. 2013; Boggia et al. 2016), no differences in baseline pre-supplementation brachial BP were observed between the morning, afternoon, and evening in healthy young men in the present study. Previous studies assessing 24 h BP have reported that the amplitudes of basal resting rhythms are small (3–6 mmHg peak-to-trough) in healthy young men and women (Scheer et al. 2010). Moreover, and also contrary to the experimental hypothesis, brachial SBP was not lowered in the morning, afternoon or evening after acute BR ingestion. Numerous previous studies have assessed the potential of NO_3_^−^ supplementation to lower BP, with a lowering in brachial SBP after NO_3_^−^ consumption observed in several (Bahadoran et al. 2017; Kapil et al. 2010; Larsen et al. 2006; Siervo et al. 2013; Bailey et al. 2010), but not all, previous studies (Siervo et al. 2013; Cermak et al. 2012; Zoughaib et al. 2023; Shepherd et al. 2015; Walker et al. 2019). However, a principal original contribution of the current study was evaluating the efficacy of NO_3_^−^ supplementation to lower brachial BP at three different time points across the day and the observation that brachial SBP was not lowered in the morning, afternoon or evening after acute BR ingestion. The factors that regulate BP across the day are multifaceted (including but not limited to renal haemodynamics, the nervous system and mental/emotional stress) and highly complex (Smolensky et al. 2017), and as such it is unclear why reductions in brachial BP were not observed in the present study.

In contrast to the peripheral brachial artery SBP response, aortic SBP was lowered from the pre-supplementation baseline after BR supplementation by a similar magnitude in the morning, afternoon and evening. Lower aortic SBP after acute NO_3_^−^ supplementation has been reported in some (Pekas et al. 2021; Kukadia et al. 2019; Hughes et al. 2016; Kim et al. 2019), but not all (Floyd et al. 2019) previous studies. In studies assessing both aortic and brachial SBP, a concurrent lowering in central and peripheral SBP has been reported (Pekas et al. 2021; Hughes et al. 2016; Kim et al. 2019), however, some studies have observed a reduction in central but not peripheral SBP (Kukadia et al. 2019; Mills et al. 2020). In contrast with the findings of the current study, both central and peripheral SBP have been reported to be lowered after acute NO_3_^−^ supplementation in the morning (Pekas et al. 2021; Hughes et al. 2016; Kim et al. 2019), with studies reporting a greater effect on central than peripheral SBP not specifying the time of the day the assessments were completed (Kukadia et al. 2019; Mills et al. 2020). Both the lowering of central SBP and the increases in plasma [NO_2_^−^] after BR ingestion were consistent across timepoints in the current study. Therefore, while previous studies have reported a strong agreement between plasma [NO_2_^−^] and brachial SBP after acute NO_3_^−^ supplementation, the current study suggests that central, but not brachial, SBP is modulated by circulating plasma [NO_2_^−^]. The mechanisms for the lowering of brachial SBP after NO_3_^−^ ingestion have been considered to be linked to the reduction of circulating plasma NO_2_^−^ to NO leading to elevated cyclic guanosine monophosphate signalling leading to vasodilation (Kapil et al. 2020, 2010). However, NO_2_^−^ can directly elicit vasodilation via s-nitrosylation (Bryan et al. 2005). In addition to increasing plasma [NO_2_^−^], acute BR ingestion has been demonstrated to increase plasma S-nitrosothiol concentrations (Abu-Alghayth et al. 2021) which have been suggested to more closely reflect the improvement in vascular function after NO_3_^−^ ingestion than plasma [NO_2_^−^] (Pinheiro et al. 2015). It has also been suggested that the lowering in BP with elevated NO_2_^−^ can occur via mechanisms independent of NO-cGMP signalling, and instead related to a novel, alternative redox pathway (mediated by hydrogen peroxide, persulfides and oxidation of protein kinase G1α), which culminates in NO-independent vasorelaxation (Feelisch et al. 2020). Therefore, further research is required to assess the mechanisms for the lowering in SBP after acute BR ingestion in humans and the extent to which this may differ in the control of central and peripheral SBP.

Acute BR ingestion largely did not modulate indices of arterial stiffness in the current study. Specifically, AP and AI were unaltered after BR ingestion in the morning or evening, but there was a small lowering in AP after BR ingestion in the afternoon. The clinical relevance of this finding in healthy young normotensive males is unclear (Wojciechowska et al. 2006). The general lack of improvement in pulse wave variables after acute NO_3_^−^ ingestion is consistent with most (Pekas et al. 2021; Kim et al. 2019; Liu et al. 2013), but not all (Hughes et al. 2016), previous studies reporting the effects of BR on AP and AI in the morning. The current study extends these previous observations by assessing the effects of BR supplementation on these variables in the afternoon and evening. It is possible that chronic NO_3_^−^ supplementation is required to improve pulse wave variables (Li et al. 2020) since arterial remodelling, including changes in the timing and/or magnitude of reflected waves from the peripheral arterial tree, may be necessary to elicit changes in central haemodynamics. Longer term NO_3_^−^ supplementation and the potential for greater overall NO exposure could positively modulate endothelial homeostasis (Carlström et al. 2018), and vascular gene expression to support vascular function (Rammos et al. 2015). In turn, such effects could contribute to lower vascular resistance and associated pulse wave variables. Therefore, our findings suggest that acute BR ingestion is more likely to lower central versus peripheral SBP, and to not influence indices of arterial stiffness in healthy young males. This finding is consistent with research in pre-diabetic and diabetic individuals after daily ingestion of dietary NO_3_^−^ for 6 months (Faconti et al. 2019). Although exact mechanisms of action are unclear, lowered SBP yet unaltered arterial stiffness with NO_3_^−^ consumption has been postulated to be due in part to increased venodilation leading to decreased preload (Mills et al. 2020).

### Exercise performance

There were no changes in high-intensity cycling TTE between the morning, afternoon and evening timepoints after PL ingestion in the current study. This observation conflicts with previous studies reporting diurnal variation in maximal voluntary contractions (Chtourou et al. 2012; Martin et al. 1999), Wingate test performance (Chtourou et al. 2012, 2011; Hammouda et al. 2012; Lericollais et al. 2009; Souissi et al. 2004) and exhaustive severe-intensity cycling exercise (Hill 2014), with performance typically peaking in the afternoon and being lowest in the morning. Acute ingestion of BR providing ~ 13 mmol NO_3_^−^ did not improve TTE during high-intensity cycling in the current study. While the existing literature generally supports a small but significant effect of NO_3_^−^ supplementation to improve performance across a range of exercise settings (Senefeld et al. 2020), and acute NO_3_^−^ supplementation has previously been reported to enhance performance during continuous high-intensity exercise tests (Wylie et al. 2013), there are also previous studies reporting no ergogenic effects in such settings (Cocksedge et al. 2020). It has previously been reported that sprint cycling performance is impaired in the morning compared to the afternoon and that acute BR ingestion (providing ~ 6.5 mmol NO_3_^−^) can improve sprint cycling performance in the morning such that it is not different from the afternoon (Dumar et al. 2021). However, a limitation of that study was the lack of an appropriate placebo supplement to ensure the participants were blinded to the treatment conditions. The current study expands on this previous study by indicating that acute BR ingestion did not improve performance compared to PL in the morning, afternoon or evening. The lack of an ergogenic effect in the current study is unlikely to be due to an insufficient NO_3_^−^ dose (Wylie et al. 2013). There is some evidence to suggest that NO_3_^−^ supplementation may be more likely to improve continuous high-intensity exercise performance after multiple-day supplementation, due to the greater time course needed to increase muscle [NO_2_^−^] (Kadach et al. 2023; Gilliard et al. 2018; Wylie et al. 2019) and subsequently impact skeletal muscle contractile function (Cermak et al. 2012; Jones et al. 2018), which may account for the lack of ergogenic effect of acute BR ingestion in the current study.

### Potential implications and experimental considerations

The findings of the current study suggest that acute BR supplementation is more likely to lower central SBP than brachial artery SBP across the course of the day. Whilst the measurement of brachial artery BP is well-established and provides strong clinical prognostic value, the importance of assessing central aortic BP and indices of aortic wave reflections has been clearly established in recent years (McEniery et al. 2014; Siervo et al. 2013). Indeed, the coronary arteries are exposed to central rather than peripheral pressures, which may account for observations that cardiovascular events may be more closely related to central pressures (McEniery et al. 2014). Moreover, the greater effect of BR ingestion on lowering central compared to brachial SBP is consistent with some studies reporting that central SBP is more likely to respond to antihypertensive treatments than brachial SBP (McEniery et al. 2014). The clinical relevance of the observed reductions in central SBP in healthy young men is currently unknown and warrants additional investigation. However, central SBP readings > 125 mmHg are associated with a significant increase in atherosclerotic cardiovascular outcomes, and for every 10 mmHg increase in central SBP the risk of an adverse cardiovascular outcomes increases by 11.7% (Kwon et al. 2022). This is important since resolving the time of day that administration of antihypertensive interventions elicits the optimal effects on cardiovascular health and cardioprotection remains unclear and an active area or research in cardiovascular medicine (Mackenzie et al. 2022; Hermida et al. 2010).

### Limitations

A challenge of administering NO_3_^−^ acutely is that the second-pass metabolism of NO_3_^−^ delays the attainment of peak plasma [NO_2_^−^] until ~ 2–4 h post ingestion, and assessments of BP and exercise performance are recommended to take place upon attainment of peak plasma [NO_2_^−^] (Wylie et al. 2013). To accommodate for the slow plasma [NO_2_^−^] pharmacokinetics after NO_3_^−^ ingestion, a limitation of the current study is that BP, vascular function and exercise performance measures were assessed early afternoon (~ 12:00–13:00), mid-afternoon (~ 16:00–17:00) and early evening (~ 19:00–20:00) after, respectively, ingesting BR in the morning (~ 09:00), early afternoon (~ 13:00) and mid-afternoon (~ 16:00). Therefore, the morning BP surge was not assessed in the current study, and the exercise test may not have been conducted early enough in the day to detect previously reported morning decrements in performance. Moreover, research in chronobiology has investigated the effect of chronotype; an individual’s predisposition towards morningness and eveningness, on responses to exercise (Vitale and Weydahl 2017). Studies have shown that circadian rhythms of physiological variables such as temperature are shifted dependent on chronotype characterisation, with biological rhythms in morningness types showing earlier peaks and troughs compared to eveningness types (Baehr et al. 2000; Bailey and Heitkemper 2001). Since chronotype was not characterized for the participants in the current study, this may have contributed to the observation of marked diurnal variability in exercise tolerance in the current study.

## Conclusion

In young healthy males, we observed no significant circadian variability in SFR, salivary pH, salivary and plasma [NO_3_^−^] and [NO_2_^−^], brachial or central BP or high-intensity exercise TTE across the non-supplemented baseline assessments. Acute NO_3_^−^-rich BR consumption resulted in similar increases in salivary and plasma [NO_3_^−^] and [NO_2_^−^] and reductions in central SBP in the morning, afternoon, and evening. In contrast, brachial SBP was unchanged following BR supplementation in the morning, afternoon, and evening and TTE was not improved at any of the timepoints assessed after BR ingestion. These findings improve our understanding of the effect of BR supplementation on BP, vascular function and exercise performance and suggest that central SBP is consistently lowered across the day after BR supplementation in healthy adults.

## Data Availability

Data are available upon reasonable request.

## Abbreviations

AFT: Afternoon
BP: Blood pressure
BR: Nitrate-rich beetroot juice
EVE: Evening
GET: Gas exchange threshold
NO: Nitric oxide
NO_2_^−^: Nitrite
NO_3_^−^: Nitrate
MORN: Morning
PAP: Peak aerobic power
PL: Nitrate-depleted beetroot juice
SBP: Systolic blood pressure
SFR: Salivary flow rate
TTE: Time to exhaustion
$\dot{V}{\text{CO}}_{{\text{2}}}$: Carbon dioxide production
$$\dot{V}{\text{E}}$$: Expired ventilation
$$\dot{V}{\text{O}}_{{\text{2}}}$$: Oxygen consumption
$\dot{V}{\text{O}}_{{{\text{2peak}}}}$: Peak aerobic capacity
